# Serum calcium, alkaline phosphotase and hemoglobin as risk factors for bone metastases in bladder cancer

**DOI:** 10.1371/journal.pone.0183835

**Published:** 2017-09-13

**Authors:** Ping Huang, Min Lan, Ai-Fen Peng, Qing-Fu Yu, Wen-Zhao Chen, Zhi-Li Liu, Jia-Ming Liu, Shan-Hu Huang

**Affiliations:** 1 Department of Nutrition, the First Affiliated Hospital of Nanchang University, Nanchang, P.R. China; 2 Department of Orthopaedic Surgery, the First Affiliated Hospital of Nanchang University, Nanchang, P.R. China; 3 School of Humanities, Jiangxi University of Traditional Chinese Medicine, Nanchang, P.R. China; University of South Alabama Mitchell Cancer Institute, UNITED STATES

## Abstract

Early detection of bone metastases is helpful for the treatment of bladder cancer (BC). In this study, we investigated the potential risk factors for bone metastasis in newly diagnosed patients with BC. A total of 902 patients diagnosed with BC between January 2000 and August 2016 were retrospectively reviewed. Of these patient, 50 (5.5%) were identified with bone metastasis. The serum levels of alkaline phosphatase (ALP) and calcium were significantly higher in patients with bone metastases than those without bone metastases (*P* = 0.015 and *P*<0.001). And the concentration of hemoglobin (HB) was significant lower in bone metastatic patients compared with non bone metastatic patients (*P* = 0.009). Multivariate logistic regression analysis indicated that ALP, HB and calcium were independent risk factors for bone metastases in patients with BC. The cut off values of ALP, HB and calcium were 116 U/L, 37.5g/L and 2.54 mmol/L according to the receiver operating characteristic (ROC) curves analysis. And combined ALP, HB with calcium had the highest diagnostic accuracy for predicting bone metastases in BC patients (AUC = 0.760, *P*<0.001). Therefore, for newly diagnosed patients with BC, the concentrations of ALP >116 U/L, HB <37.5 g/Land calcium >2.54 mmol/L were the risk factors for developing bone metastases. Combined ALP, HB with calcium was more useful to diagnose the bone metastases.

## Introduction

Bone is a relatively frequent site of symptomatic metastasis in patients with solid tumors, such as lung, prostate, breast and renal cancers [1], but not common in patients with bladder cancer (BC). The prevalence of BC was steadily increasing in China over the recent years [2]. And the presence of bone metastases is correlated with skeletal complications and decreased survival [3]. Therefore, early diagnosis of bone metastases from BC is benefit for timely intervention of it and prevention of pathologic bone fracture.

Diagnosis of patients with bone metastases primarily relies on imaging study. And bone scintigraphy is routinely used for the detection of bone metastases. However, it lacks specificity on detection of skeletal lesions, and the value of bone scintigraphy for detecting cancer progression was questionable on the basis of cost-effectiveness [4].

Since routinely used tumor markers in BC can’t accurately predict the risk of metastasizing to bone [5], other risk factors for bone metastases are required. Many studies have attempted to identify risk factors for tumor progression, prognosis and respondence to treatment in the patients with bone metastases [6, 7]. However, to our knowledge, risk factors for bone metastases from BC have been examined in few studies and still unclear.

The present study was performed to investigate the correlation between clinical parameters and bone metastases in patients with BC, and to identify the independent risk factors for detecting bone metastases.

## Materials and methods

### Patient selection

This study was approved by the ethics committee of the First Affiliated Hospital of Nanchang University, and informed consents were harvested from all patients. A retrospective study, including 1229 patients with BC admitted to our hospital between January 2000 and September 2016, was carried out. For the diagnosis, the confirmation as primary BC was based on the histopathologic analysis of specimens. Bone metastases were diagnosed by bone scan. If necessary, local computed tomography (CT) scan and magnetic resonance imaging (MRI) would be used to make sure of the diagnosis. Patients who presented with a history of bone metabolic disorders, significant hematologic disorders, hyperparathyroidism, severe liver and/or renal insufficiency, and other malignant tumors were excluded from this study. In addition, patients who had other distant metastases, such as liver and lung, were not included in the study.

### Data collection

Patients’ demographics and clinical-pathological parameters, including age at diagnosis of the primary tumor, gender, histopathologic types, laboratory findings, such as alkaline phosphate (ALP), hemoglobin (HB) and serum calcium levels were retrospectively reviewed. The biomarkers were measured in sera obtained from peripheral venous blood. The measurement of serum ALP is total ALP and serum calcium is ionized calcium in this study. All the measurement was conducted by the Department of Nuclear Medicine of the hospital. All of the data were collected at the time of patients firstly diagnosis at our hospital before giving any treatment. The concentrations of ALP, HB and calcium were measured in serum samples from patients with BC.

### Statistical analysis

All analysis was performed by SPSS Version 22 (SPSS Inc. Chicago IL). Qualitative variables were expressed as numbers and percentages, and were analyzed by the *Chi*-square test. Quantitative variables were reported as means ± standard deviation and analyzed by *Student’s t* test. Multivariate logistic regression model was used to identify the independent risk factors for bone metastasis in BC. Receiver operating characteristic (ROC) curve was used to assess the predictive power of risk factors for bone metastases. Statistical significance was set as *P* value less than 0.05.

## Results

### Patient demographics

A total of 902 patients were included in this study and 327 patients were excluded according to the inclusion and exclusion criteria. **Table 1**showed the demographics of patients with BC. Of these patients, 81.6% were males and 18.4% were females. The majority of histopathologic types were transitional cell carcinoma, which accounts for 91.2% (823 cases) of the patients. A total of 50 cases (5.5%) developed bone metastases in this group, and 82% (41 cases) of them were males. Of the 50 patients with bone metastases, 31 (62%) were osteolytic lesion, 14 (28%) were osteoblastic lesion and 5 (10%) were mixed bone lesion.

**Table 1:** Demographics of patients with bladder cancer.

Characteristics	Patients, n (%) (N = 902)
**Age (years)**	
Median	66
Range	19–93
**Gender**	
Male	736 (81.6)
Female	166 (18.4)
**Histopathologic types, n (%)**	
Transitional cell carcinoma	823 (91.2)
Squamous cell carcinomas	48 (5.3)
Adenocarcinomas	28 (3.1)
Other	3 (0.3)
**Number of patients with bone metastases**	50(5.5)
Male	41 (82)
Female	9 (18)

### The distribution of bone metastases from bladder cancer

For the patients with bone metastases, the most commonly affected sites were the pelvis (68%), followed by the spine (cervical 12%, thoracic 38% and lumbar 34%) and ribs (24%). The humerus was the least metastatic sites in these patients (**Table 2**).

**Table 2:** The distribution of bone metastases in patients with bladder cancer.

Site of bone metastases	Patients, n (%)(N = 50)
** Pelvis**	34 (68)
** Spine**	
Cervical	6 (12)
Thoracic	19 (38)
Lumbar	17 (34)
** Ribs**	12 (24)
** Femur**	11 (22)
** Skull**	7 (14)
** Clavicle**	5 (10)
** Sternum**	4 (8)
** scapula**	4 (8)
** Humerus**	3 (6)

### Risk factors for bone metastases in patients with bladder cancer

Based on the analysis, there were no statistically significant differences on age, gender and histopathologic type between BC patients with and without bone metastasis (*P* >0.05, respectively). Also, no statistical differences were noted for the biomarkers of AFP, CEA, CA125, CA153, CA199 and CA724 between the two groups (*P* >0.05, respectively). However, the serum concentrations of ALP and calcium were significantly higher in patients with bone metastases compared to those without bone lesions (*P* = 0.015 and *P*<0.001). And the HB level was significantly lower in bone metastatic patients than those in non bone metastatic patients (*P* = 0.009) (**Table 3**). Multivariate logistic regression analysis indicated that ALP (OR = 0.007, *P*<0.001), HB (OR = -0.032, *P* <0.001) and calcium (OR = 2.569, *P* <0.001) were the independent risk factors correlated with bone metastases in patients with BC (**Table 4**).

**Table 3:** The differences on the clinical-pathological parameters between patients with and without bone metastases.

Characteristics	BM (n = 50)	NBM (n = 852)	*P*
**Age (years)**	66.40±11.14	64.74±11.93	0.337
**Gender, n (%)**			0.940
Male	41 (82)	695 (81.6)	
Female	9 (18)	157 (18.4)	
**Histopathologic types, n (%)**			0.662
Transitional cell carcinoma	44 (88)	779 (91.4)	
Squamous cell carcinomas	3 (6)	45 (5.3)	
Adenocarcinomas	3 (6)	25 (3.0)	
Other	0	3 (0.3)	
**AFP (ug/L)**	2.68±1.95	2.49±1.46	0.719
**CEA (ug/L)**	16.40±33.81	10.41±53.19	0.550
**CA125 (u/ml)**	147.09±313.25	57.56±185.19	0.175
**CA153 (u/ml)**	43.65±76.60	15.70±21.03	0.181
**CA199 (u/ml)**	153.89±284.05	49.80±152.47	0.098
**CA724 (u/ml)**	22.33±38.41	12.13±41.97	0.538
**ALP (u/L)**	244.18±468.00	76.55±47.44	0.015
**HB (g/L)**	97.16±25.37	115.63±24.46	<0.001
**Calcium (mmol/L)**	2.40±0.39	2.24±0.23	0.009

BM: Bone metastases group; NBM: Non bone metastases group; ALP: alkaline phosphate; HB: hemoglobin.

**Table 4:** The risk factors for detecting bone metastases in patients with bladder cancer.

Factors	B	OR	OR (95% CI)	*P*
**ALP**	0.007	1.007	1.004–1.010	<0.001
**HB**	-0.032	0.968	0.957–0.980	<0.001
**Calcium**	2.569	13.049	3.836–44.384	<0.001

B: coefficient of regression, OR: odds ratio, CI: confidence interval.

### The diagnostic accuracy of risk factors for predicting bone metastases

**Fig 1:**
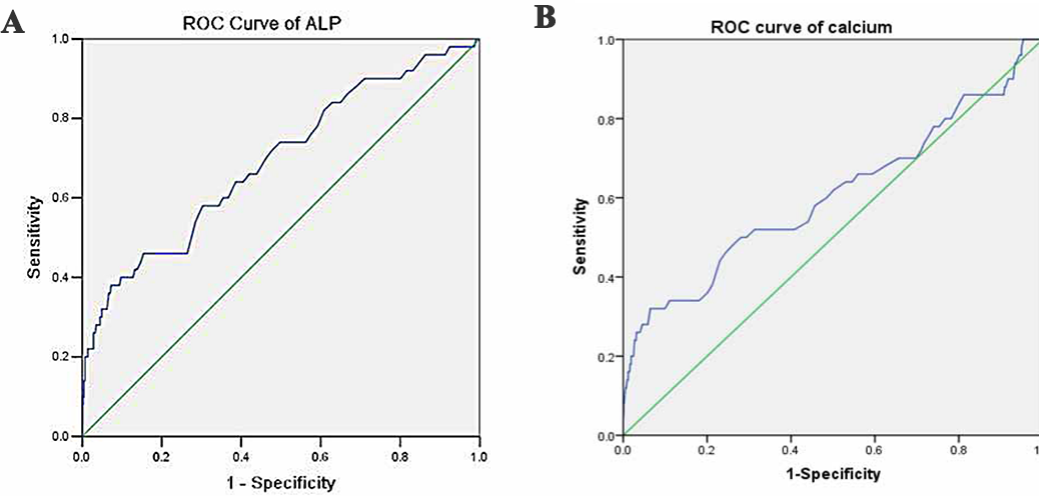
Receiver operating characteristics (ROC) curves of single risk factor for detecting bone metastases in patients with bladder cancer. (A). the ROC of ALP. (B). the ROC of calcium.

**Fig 2:**
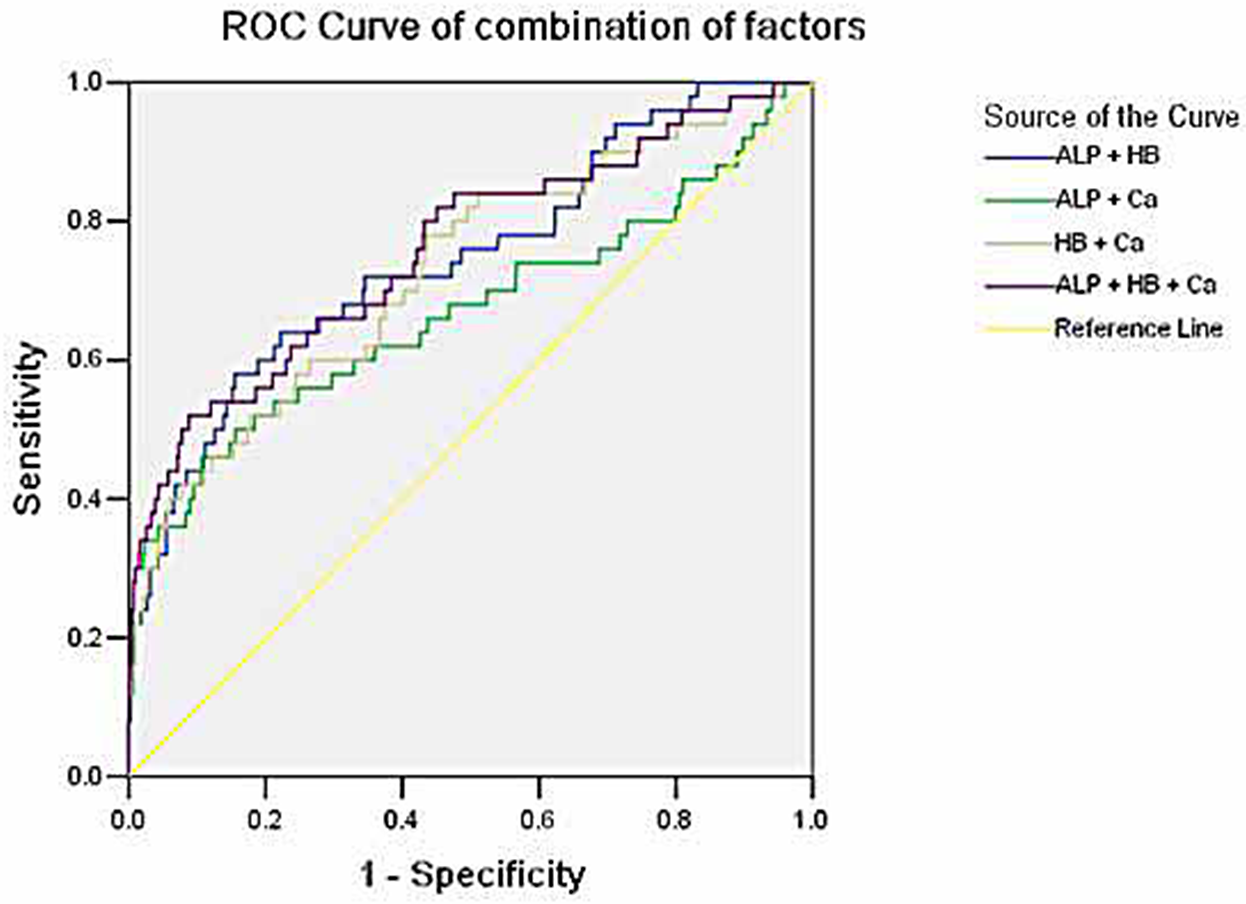
Receiver operating characteristics curves of different combined risk factors for diagnosing bone metastases in bladder cancer patients.

**Fig 1**showed the ROC curves of single factor for predicting the risk of developing bone metastasis in patients with BC. Based on the analysis, the concentration of ALP had the highest diagnostic accuracy among the three factors for bone metastases in BC [area under the curve (AUC) = 0.691, *P*<0.001], with a sensitivity of 38% and specificity of 92.7%. The cut-off values of ALP, HB and calcium were 116U/L, 37.5g/L and 2.535mmol/L, respectively (**Table 5**). In order to identify the accuracy of combined factors for diagnosing bone metastases, we analyzed the ROC curves for different combinations of risk factors. The results indicated that combined ALP, HB with calcium had the highest diagnostic accuracy for predicting bone metastases in BC (AUC = 0.760, *P*<0.001) (**Table 5 and Fig 2**).

**Table 5:** The cut off values, sensitivities and specificities of risk factors for predicting bone metastasis in patients with bladder cancer.

Factors	Cut-off value	Sensitivity (%)	Specificity (%)	AUC	*P*
**ALP (u/L)**	116	38	92.7	0.691	<0.001
**HB (g/L)**	37.5	100	0.4	0.299	<0.001
**Calcium (mmol/L)**	2.535	32	93.5	0.606	0.012
**ALP + HB**	-	58	84.5	0.747	<0.001
**ALP + CA**	-	46	89.3	0.670	<0.001
**HB + CA**	-	78	56.3	0.730	<0.001
**ALP+HB+CA**	-	52	91.2	0.760	<0.001

ALP: alkaline phosphatase, HB: hemoglobin, CA: Calcium, AUC: area under the curve.

## Discussion

Bone metastasis is a common feature of advanced genitourinary malignancies and a prominent cause of morbidity and mortality. Clinical manifestations of bone metastasis include pain, pathologic fractures and spinal cord compression [8], which will significantly decrease the quality of patient’s life. Fortunately, some studies showed the application of bisphosphonates would prevent and delay bone metastases in solid tumors [9]. Given that, early detection of bone metastases will significantly improve the treatment of BC. Identifying available and inexpensive risk factors is a meaningful benefit for timely intervention and prevention of bone metastases in BC. Thus, we carried out this study to determine the risk factors from patients’ clinical-pathological parameters for bone metastases.

For the rate of bone metastases from BC, previous study reported that 4–14.5% of patients with BC developed bone metastases [10, 11]. In the present study, 5.5% of the patients were found to be bone metastases. And the most commonly metastatic sites of bone were the pelvis (68%), followed by the spine in our study, which were in accordance with Taher et al’s reports [11]. In Taher et al’s study, the pelvic bone and spine were also found to be the most frequent sites affected by metastases in BC patients.

Based on the analysis of this study, high concentrations of ALP and calcium, and low HB level were found to be the independent risk factors for predicting bone metastases in patients with BC. ALP is synthesized in the liver and osteoblasts, and has been used as a specific biomarker for liver and bone. Previous studies revealed that ALP was a biomarker for the presence of bone metastases before their appearance on the bone scintigraphy [6, 12, 13]. In patients with BC, ALP was found to be a significant prognostic factor in bladder cancer with definitive radiotherapy [14]. In this study, the results showed that the serum level of ALP was correlated with bone metastases in BC, with a cut off value of 116U/L. It meant that ALP >116U/L was a risk factor for developing bone metastasis in patients with BC. Also, we found that 28% of bone metastases were osteoblastic lesion and 10% were mixed bone lesion in these patients. This can be interpreted why ALP were increased in BC patients with bone metastases.

Kawai et al [15] revealed that HB may promote the first step of cancer cells to adhere to bone marrow during the process of bone metastasis from prostate cancer. In the current study, we found the concentration of HB was an independent factor for bone metastasis in BC. The cutoff values of it was 37.5g/L, which suggested that the serum hemoglobin level < 37.5g/L could be used to identify patients with BC at a higher risk for bone metastasis. However, the specificity of HB for diagnosing bone metastases was relatively low (0.4%).

For human body, the majority of calcium is stored in bone, and the concentration of it is under strictly hormonal regulation at the level of resorption in the kidney, mobilization from the skeleton and intestinal absorption [16]. Study showed that serum calcium of patients with lung cancer maybe a valuable biomarker to diagnose bone metastasis in early stage [17]. Additionally, high expression level of calcium was a potential new factor for predicting the risk of bone metastases, and it promoted bone metastases from solid cancer via enhancing the expression of calcium-sensing receptor [18]. In line with previous studies, the concentration of calcium was increased in patients with bone metastases and identified as an risk factor for bone metastasis in patients with BC. The cutoff value of it was 2.54mmol/L. It indicated that the concentration of calcium > 2.54mmol/L was a risk factor for bone metastases in BC. Additionally, we found that 62% of bone metastases in this study were osteolytic lesion. This is why the concentration of calcium was increased in BC patients with bone metastases.

In the present study, we also analyzed the diagnostic accuracy of combined risk factors for predicting bone metastases in patient with BC. Compared to the single factor of ALP, HB and calcium, combined ALP, HB with calcium had a higher accuracy for predicting bone metastases in BC. It suggested that combination of risk factors appeared to be more accurate for predicting bone metastasis and may provide important information for patients with BC.

Although the results of this study were interesting, there were still some limitations in it. First of all, this is a retrospective study and we just collected the data at the time of primary diagnosis. Some follow-uped information, such as time to bone metastasis and survival duration, were insufficient and not reported in this study. Second, the study was conducted in a single medical center with a relative small sample, which may not present the characteristics of the whole population with BC. Also, we were failed to perform the analysis based on tumor categories. Third, most of the patients didn’t report the stage of tumor due to the retrospective methodology. And we couldn’t make clear of the risk factors for bone metastases according to the tumor stage. Fourth, the sensitivities and specificities of these risk factors for predicting bone metastases in BC were not high. Further study with large sample and multi-center investigation is necessary to make sure the efficacy of these risk factors for predicting bone metastases in BC.

In conclusion, based on a large population analysis, the present study suggested that the serum concentrations of ALP, HB and calcium were independent risk factors for bone metastases in patients with BC. For newly diagnosed patients, the values of ALP >116U/L, HB <37.5g/L and calcium >2.535mmol/L could be used as additional factors for determining the risk of developing bone metastases. And combined ALP, HB with calcium had the highest diagnostic accuracy for predicting bone metastases. However, the validation of these factors in clinical practice need further study to confirm.

## Supporting information

S1 File The original data of patients included in this study. (XLSX)
